# Impact of Different Types of Lymphadenectomy Combined With Different Extents of Tumor Resection on Survival Outcomes of Stage I Non-small-cell Lung Cancer: A Large-Cohort Real-World Study

**DOI:** 10.3389/fonc.2019.00642

**Published:** 2019-07-24

**Authors:** Weidong Wang, Dongni Chen, Kexing Xi, Yongqiang Chen, Xuewen Zhang, Yinsheng Wen, Zirui Huang, Xiangyang Yu, Gongming Wang, Rusi Zhang, Lanjun Zhang

**Affiliations:** ^1^Department of Thoracic Surgery, Sun Yat-sen University Cancer Center, Guangzhou, China; ^2^Department of Thoracic Surgery, School of Medicine, The First Affiliated Hospital, Zhejiang University, Hangzhou, China; ^3^State Key Laboratory of Oncology in South China, Collaborative Innovation Center for Cancer Medicine, Sun Yat-sen University Cancer Center, Guangzhou, China; ^4^Department of Colorectal Surgery, National Cancer Center/Cancer Hospital, Chinese Academy of Medical Sciences and Peking Union Medical College, Beijing, China; ^5^Department of Oncology, Sun Yat-sen University Cancer Center, Guangzhou, China

**Keywords:** non-small-cell lung cancer, lymphadenectomy, prognosis, real-world study, propensity score matched

## Abstract

**Background:** To investigate the prognostic impact of different types of lymphadenectomy with different extents of tumor resection on the outcomes of stage I non-small-cell lung cancer (NSCLC).

**Methods:** Patients were classified into lobectomy and sublobectomy groups, and then each group was subdivided according to the types of lymphadenectomy. The end points of the study were overall survival (OS) and disease-free survival (DFS). Propensity score matched (PSM) comparative analysis and univariate and multivariate Cox regression analyses were performed.

**Result:** A total of 1,336 patients were included in the current study. Lobectomy was associated with better OS and DFS. In the lobectomy group, lobectomy with bilateral mediastinal lymphadenectomy (BML) was associated with better OS than lobectomy with systematic nodal dissection (SND) or lobe-specific systematic node dissection (L-SND). Lobectomy with SND or L-SND was associated with better OS than lobectomy with systematic nodal sampling (SNS) or selected lymph node biopsy (SLNB). Additionally, lobectomy with BML or SND was associated with better DFS than lobectomy with L-SND or SNS or SLNB. After PSM, compared with lobectomy with SNS or SLNB, lobectomy with SND resulted in more favorable OS and DFS. There was no survival difference between different types of lymphadenectomy for patients who underwent sublobectomy. A multivariable analysis revealed independent associations of lobectomy with BML or SND with better OS and DFS compared with those of lobectomy with SNS or SLNB.

**Conclusion:** This study reveals an association of lobectomy with more systematic and complete lymph node dissection, such as BML or SND, with better prognosis in stage I NSCLC patients.

## Introduction

Non-small-cell lung cancer (NSCLC) is the malignancy with the highest morbidity and mortality rates worldwide (1). However, improved radiologic imaging and widespread low-dose computed tomography screening have led to increased detection of early-stage (stage I) NSCLC (2). For surgically resectable lung cancer, surgery is the best therapeutic option. Additionally, lymph node (LN) dissection during surgery is essential. For these reasons, determining the appropriate model of resection of original tumors and LN dissection has attracted more attention.

The European Society of Thoracic Surgeons (ESTS) guidelines (3) for LN dissection in NSCLC classify intraoperative LN dissection into the following five categories: (1) selected lymph node biopsy (SLNB), (2) systematic nodal sampling (SNS), (3) systematic nodal dissection (SND), (4) lobe-specific systematic node dissection (L-SND), and (5) extended lymph node dissection (ELND). Additionally, lobectomy with SND is considered to be a standard therapy for patients with NSCLC, but for some early-stage patients, sublobectomy or SNS is also acceptable (4).

Sublobar resection has been indicated to result in a similar survival rate for early-stage NSCLC patients compared with that of lobectomy, and it can retain more pulmonary function (5). However, some researchers hold the opposite view (6), and they believe that sublobectomy results in an inferior survival rate. Several previous studies have indicated that among patients who underwent sublobectomy, whether LN dissection was performed did not influence the survival outcomes (7). However, there are also some studies reporting that LN dissection can result in a better prognosis even in patients who have undergone sublobectomy (8). The need for SND has also been questioned. Whether SND compared with SNS can provide more accurate staging and survival benefit or lead to more complications in stage I NSCLC patients has not yet been confirmed (9, 10). Some studies have also shown that L-SND results in the same survival rate as that of SND, although it decreases the duration of the surgery and incidence of postoperative complications. However, some studies have found that L-SND may be ineffective in some N2-positive patients, influencing the pathological grading and adjunctive therapy (11, 12).

Therefore, in our study, we used a large cohort of patients to compare the outcomes of different LN dissection models combined with different extents of resection in stage I NSCLC patients to formulate guidelines regarding the extent of tumor resection and lymphadenectomy.

## Materials and Methods

### Patients

This study initially included 2,102 consecutive cases consisting of clinical and pathological stage I NSCLC patients who underwent surgical treatment between 1999 and 2014 in the Department of Thoracic Surgery in Sun Yat-sen University Cancer Center. This study was approved by the Institutional Review Board of Sun Yat-sen University Cancer Center.

**Figure 1:**
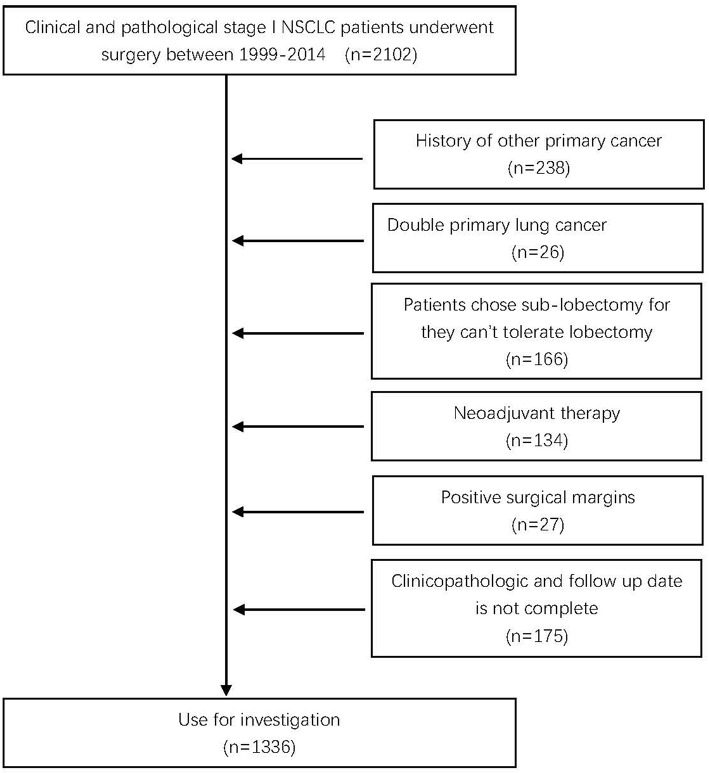
Inclusion and exclusion criteria. NSCLC, non-small-cell lung cancer.

All patients were confirmed to be pathological stage I according to the 8th edition of the American Joint Committee on Cancer (AJCC) lung cancer staging classification (13) and met the following criteria: (1) primary NSCLC; (2) preoperatively considered node negative; and (3) pathologic stage was T1a-2aN0M0. The exclusion criteria were as follows: (1) a history of other primary cancer; (2) double primary lung cancer; (3) the patient received neoadjuvant therapy; (4) positive surgical margins; (5) the clinicopathologic and follow-up data were not complete; and (6) patients chose sublobectomy because they could not tolerate lobectomy (Figure 1). Finally, 1,336 patients were included in further analysis.

### Study End Points

The outcomes of this study included overall survival (OS) and disease-free survival (DFS). The latest follow-up of the current study was performed on October 15, 2018.

### Patients Grouping

The location and station of mediastinal LNs were based on the International Association for the Study of Lung Cancer LN map (14). For analytical purposes, we divided the patients into two major groups (Group Lobe: for those patients who underwent lobectomy, biolobectomy, or pneumonectomy; and Group Sublobe: for those patients who underwent wedge resection or segmentectomy) and then into five or three smaller groups, respectively, according to the LN dissection as follows: Group Lobe: A, patients with SLNB or SNS; B, patients with SND; C, patients with L-SND; D, patients without any LN dissection; and E, patients with bilateral mediastinal lymphadenectomy (BML); and Group Sublobe: F, patients with SND; G, patients with SLNB or SNS; and H, patients without any LN dissection (Table 1). The definitions of all options in the intraoperative LN dissection model are as follows: (1) Selected LN biopsy: one or multiple suspicious LN(s) were biopsied; (2) SNS: a predetermined selection of one or more LN stations specified by the surgeon; (3) SND: all the mediastinal tissue containing the LNs is dissected and removed systematically within anatomical landmarks, and at least three mediastinal nodal stations (but always subcarinal) should be excised, and the hilar and the intrapulmonary LNs are dissected as well; (4) L-SND: the mediastinal tissue containing specific LN stations are excised, depending on the lobar location of the primary tumor; and (5) BML: bilateral mediastinal LN dissection is performed through cervical mediastinoscopy (3).

**Table 1:** Description of the 1,336 patients.

**Group**	**Subgroup**	**Description**
Group Lobe	A (*n* = 346)	Lobectomy with selected lymph node biopsy or sampling
	B (*n* = 328)	Lobectomy with systematic lymph node dissection
	C (*n* = 577)	Lobectomy with lobar-selective lymph node dissection
	D (*n* = 13)	Lobectomy without any lymph node dissection
	E (*n* = 28)	Lobectomy with bilateral mediastinal lymphadenectomy
Group Sublobe	F (*n* = 3)	Sublobectomy with systematic lymph node dissection
	G (*n* = 13)	Sublobectomy with randomly lymph node dissection or sampling
	H (*n* = 28)	Sublobectomy without any lymph node dissection

A propensity score matched (PSM) comparative analysis was performed to control the non-random variables among groups. We adjusted for potential differences between Group A (patients with SLNB or SNS) and Group B (patients with SND) (1:1 match), and finally 468 patients (234 in each group) were included in the PSM analysis. We generated a propensity score for the matched groups using logistic regression based on the patients' potential confounding baseline characteristics, including age, tumor size, tumor location, and surgical approach. We next created a balanced cohort using an optimized performance-matching algorithm with a caliper setting of 0.02. The procedure was conducted using SPSS 21.0.

### Statistical Analysis

Categorical variables were calculated using the χ^2^ test, and continuous variables were analyzed using the *t*-test. All end points were estimated using the Kaplan–Meier method and compared using the log-rank test. Multivariable survival analyses were performed using the Cox proportional hazards model to identify prognostic factors for OS and DFS. For all analyses, a two-sided *P* < 0.05 was considered statistically significant. Hazard ratios (HRs), 95% confidence intervals (CIs), and *P*-values for each variable were determined using SPSS 21.0 software (IBM, Armonk, NY), and survival curves were drawn using Prism 7.0 (GraphPad software, La Jolla, CA).

## Results

### Patient Characteristics

In total, 44 patients underwent sublobectomy, and 1,292 patients underwent lobectomy, including 346 (25.9%) patients in Group A, 328 (24.6%) in Group B, 577 (43.2%) in Group C, 13 (1.0%) in Group D, 28 (2.1%) in Group E, 3 (0.2%) in Group F, 13 (1.0%) in Group G, and 28 (2.1%) in Group H. There were 822 males and 470 females in Group Lobe and 33 males and 11 females in Group Sublobe. The mean age of Group Lobe was 59.35 ± 10.1 years, and the median age was 60.0 years. In addition, in Group Sublobe, the mean age was 66.73 ± 11.9 years. Non-squamous cell carcinoma was the most common (1,002 and 40 patients, 77.6 and 90.9%, respectively) pathologic type in both groups. When the clinicopathologic characteristics were compared among groups, it was interesting to note that sex, histology, cell differentiation, smoking history, adjuvant therapy, and treatment after disease progression were well-balanced between the subgroups in both Group Lobe and Group Sublobe. Patients in Group Lobe were younger, had larger tumors, and had more LNs resected than those in Group Sublobe. Among the patients in Group Lobe, lobectomy was much more common than bilobectomy or pneumonectomy (1,234 vs. 33 and 25, respectively). Interestingly, in later procedures, there were more patients who underwent LN dissection rather than LN sampling in Group Lobe. Among Group Sublobe, most patients underwent wedge resection without any LN dissection. Notably, those who received SND in Group Sublobe underwent segmentectomy. The baseline characteristics of the patients in Group Lobe are summarized in Table 2, and those in Group Sublobe are summarized in Supplemental Table 1.

**Table 2:** Distribution of the clinicopathologic characteristics stratified by group in Group Lobe (*n* = 1,292).

**Characteristic**	**Total**	**Group A (*n* = 346)**	**Group B (*n* = 328)**	**Group C (*n* = 577)**	**Group D (*n* = 13)**	**Group E (*n* = 28)**	***P***
**Sex**							0.361
Male	822(63.6)	233(67.3)	204(62.3)	359(62.2)	10(76.9)	16(57.1)	
Female	470(36.4)	113(32.7)	124(37.7)	218(37.8)	3(23.1)	12(42.9)	
**Age(years)**							<0.001
Mean ± SD	59.35 ± 10.1	59.99 ± 10.7	58.96 ± 9.8	58.94 ± 10.0	73.77 ± 6.4	58.86 ± 7.5	
Median(min, max)	60(16, 84)	61(23, 84)	59(32, 80)	60(16, 80)	76(61, 84)	59.5(46, 74)	
**Year of procedure**							<0.001
1999–2002	78(6.0)	43(12.4)	5(1.8)	26(4.5)	4(30.8)	0	
2003–2006	155(12.0)	76(22.0)	25(7.6)	51(8.8)	3(23.1)	0	
2007–2010	372(28.8)	122(35.3)	77(23.4)	166(28.8)	4(30.8)	3(10.7)	
2011–2014	687(53.2)	105(30.3)	221(67.2)	334(57.9)	2(15.4)	25(89.3)	
**Histology**							0.061
Non-squamous cell carcinoma	1002(77.6)	267(77.2)	239(72.9)	462(80.1)	9(69.2)	25(89.3)	
Squamous cell carcinoma	290(22.4)	79(22.8)	89(27.1)	115(19.9)	4(30.8)	3(10.7)	
**Cell differentiation**							0.797
Poor–None	468	121(35.0)	126(38.3)	204(35.4)	5(38.5)	12(42.9)	
Well–Moderate	824	225(65.0)	202(61.7)	373(64.6)	8(61.5)	16(57.1)	
**Tumor size (cm)**							0.067
Mean ± SD	2.67 ± 1.0	2.77 ± 1.0	2.7 ± 1.0	2.59 ± 0.9	2.69 ± 1.0	2.61 ± 1.0	
Median(min, max)	2(0, 4)	3(0, 4)	3(1, 4)	2(0, 4)	2(1, 4)	2(1, 4)	
**Smoking history**							0.730
Yes	503(38.9)	140(40.5)	133(40.7)	217(37.6)	4(30.8)	9(32.1)	
No	789(61.1)	206(59.5)	195(59.3)	360(62.4)	9(69.2)	19(67.9)	
**Pathological T category**							0.002
T1a	57(4.3)	10(2.9)	20(6.1)	26(4.3)	0	1(3.6)	
T1b	228(17.7)	48(13.9)	52(15.9)	122(21.1)	0	6(21.4)	
T1c	204(15.8)	42(12.1)	67(20.4)	89(15.4)	1(7.7)	5(17.9)	
T2a	803(62.2)	246(71.1)	188(57.6)	341(59.1)	12(92.3)	16(57.1)	
**Pathological stage**							0.002
I A1	57(4.3)	10(2.9)	20(6.1)	26(4.3)	0	1(3.6)	
I A2	228(17.7)	48(13.9)	52(15.9)	122(21.1)	0	6(21.4)	
I A3	204(15.8)	42(12.1)	67(20.4)	89(15.4)	1(7.7)	5(17.9)	
I B	803(62.2)	246(71.1)	188(57.6)	341(59.1)	12(92.3)	16(57.1)	
**Adjuvant therapy**							0.202
Yes	188(14.6)	58(16.8)	41(12.5)	85(14.7)	4(30.8)	1(3.6)	
No	1104(85.4)	288(83.2)	287(87.5)	492(85.3)	9(69.2)	27(96.4)	
**Tumor location**							<0.001
LUL	341(26.4)	101(29.2)	84(25.5)	139(24.1)	5(38.5)	12(42.9)	
LLL	180(13.9)	73(20.8)	43(13.1)	59(10.2)	0	6(21.4)	
LL	8(0.6)	0	4(1.2)	4(0.7)	0	0	
RUL	416(32.2)	59(17.1)	119(36.5)	229(19.7)	1(7.7)	8(28.6)	
RML	116(9.0)	22(6.4)	12(3.6)	78(13.5)	3(23.1)	1(3.6)	
RLL	220(17.0)	89(25.7)	60(18.2)	66(11.4)	4(30.8)	1(3.6)	
RUML	2(0.1)	1(0.3)	1(0.3)	0	0	0	
RMLL	8(0.6)	2(0.6)	4(1.2)	2(0.3)	0	0	
RL	1(0.1)	0	1(0.3)	0	0	0	
**Surgical approach**							0.089
Lobectomy	1234(95.5)	334(96.5)	308(93.9)	552(95.7)	13(100.0)	27(96.4)	
Biolobectomy	33(2.6)	9(2.6)	15(4.6)	9(1.6)	0	0	
Pneumonectomy	25(1.9)	3(0.9)	5(1.5)	16(2.8)	0	1(3.6)	
**Number of lymph nodes resected**							<0.001
Mean ± SD	18.49 ± 11.0	11.66 ± 6.9	25.02 ± 10.2	18.09 ± 8.5	0	42.71 ± 21.3	
Median(min, max)	17(0, 125)	10(1, 43)	23(7, 79)	17(3, 57)	0	39(9, 125)	
**Treatment after progression of disease**							0.506
Yes	191(14.8)	54(15.6)	47(14.3)	83(14.4)	4(30.8)	3(10.7)	
No	1101(85.2)	292(84.4)	281(85.7)	494(85.6)	9(69.2)	25(89.3)	
**EGFR mutation**							<0.001
Negative	228(17.6)	41(11.8)	75(22.8)	107(18.5)	1(7.7)	4(14.3)	
Positive	209(16.2)	34(15.5)	53(24.2)	111(19.2)	0	11(39.3)	
Not tested	855(66.2)	271(78.3)	200(61.1)	359(62.2)	12(92.3)	13(46.4)	
**ALK mutation**							<0.001
Negative	295(22.8)	44(12.7)	83(25.2)	156(27.0)	0	12(42.9)	
Positive	9(0.7)	2(0.6)	2(0.6)	5(0.9)	0	0	
Not tested	988(76.5)	300(86.7)	243(72.1)	416(72.1)	13(100.0)	16(57.1)	

*ALK, anaplastic lymphoma kinase; cm, centimeter; EGFR, epidermal growth factor receptor; LUL, left upper lobe; LLL, left lower lobe; LL, left lung; max, maximum; min, minimum; RUL, right upper lobe; RML, right middle lobe; RLL, right lower lobe; RUML, right upper-middle lobe; RMLL, right middle-lower lobe; RL, right lung; SD, standard deviation*.

### Survival Analysis

1. Comparison of survival between the lobectomy group and sublobectomy group

**Figure 2:**
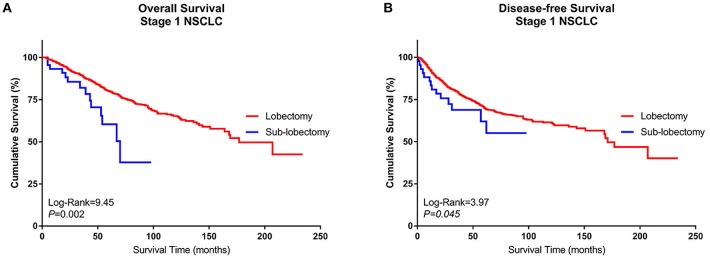
Kaplan–Meier curves of the survival estimates for patients who underwent lobectomy or sublobectomy. **(A)** Overall survival data from patients who underwent lobectomy or sublobectomy for stage I NSCLC. **(B)** Disease-free survival of patients who underwent lobectomy or sublobectomy for stage I NSCLC. NSCLC, non-small-cell lung cancer.

A Kaplan–Meier survival analysis and log-rank comparison revealed that compared with the Group Sublobe, the lobectomy group (Group Lobe) was significantly associated with better OS (log rank = 9.45, *P* = 0.002) and DFS (log rank = 3.97; *P* = 0.045) in patients with stage I NSCLC (Figures 2A,B).

2. Comparison of survival between different types of lymphadenectomy in Group Lobe

**Figure 3:**
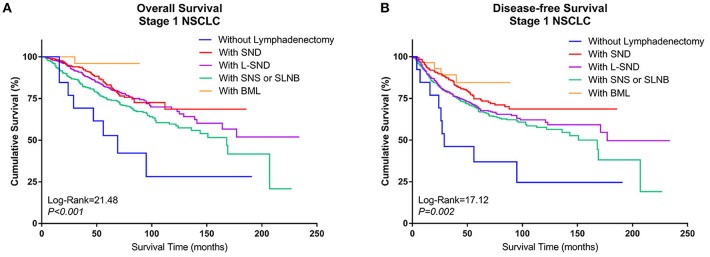
Kaplan–Meier curves of survival estimates for patients who underwent lobectomy with different types of lymphadenectomy. **(A)** Overall survival of patients who underwent lobectomy with different types of lymphadenectomy for stage I NSCLC. **(B)** Disease-free survival of patients who underwent lobectomy with different types of lymphadenectomy for stage I NSCLC. NSCLC, non-small-cell lung cancer.

When the patients who underwent lobectomy were divided into Groups A to E, it was clear that the patients who underwent lobectomy without LN dissection (Group D) had the worst OS. Among those who underwent LN dissection, patients who underwent systematic LN dissection (Group B) or L-SND (Group C) had better outcomes than patients who underwent selective LN biopsy or sampling (Group A), although the difference between Group B and Group C was not significant. Patients who underwent BML (E) had the best postoperative survival among the subgroups in Group Lobe. The 5-year survival rates were 79.5, 87.2, 85.2, 53.8, and 96.4% in Group A through Group E, respectively, and the Kaplan–Meier curves showed a significant difference in OS (log rank = 21.48, *P* < 0.001). Similar to OS, Group D had the worst DFS, while Group B and Group E had better DFS than Group C and Group A. The 5-year DFS rates were 72.8, 79.3, 72.4, 38.5, and 78.6% in Group A through Group E, respectively. The Kaplan–Meier curves also showed a significant difference in DFS among the subgroups (log rank = 17.12, *P* = 0.002) (Figures 3A,B).

3. Comparison of survival between different types of lymphadenectomy in Group Sublobe

**Figure 4:**
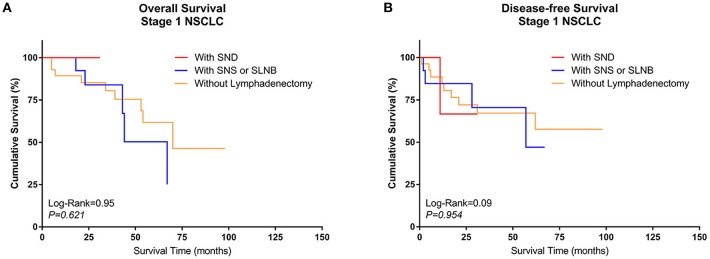
Kaplan–Meier curves of survival estimates for patients who underwent sublobectomy with different types of lymphadenectomy. **(A)** Overall survival of patients who underwent sublobectomy with different types of lymphadenectomy for stage I NSCLC. **(B)** Disease-free survival of patients who underwent sublobectomy with different types of lymphadenectomy for stage I NSCLC. NSCLC, non-small-cell lung cancer.

A similar subgroup analysis was performed in Group Sublobe. However, there were no significant differences in either OS or DFS among Group F, Group G, and Group H (*P* = 0.621 and *P* = 0.954, respectively) (Figures 4A,B).

4. Comparison of survival via Cox regression

In Group Lobe, a multivariable Cox proportional hazard model revealed independent associations of lobectomy plus systematic LN dissection, lobar-selective LN dissection, or BML with better OS (HR, 0.668; 95% CI, 0.483–0.923; *P* = 0.015; HR, 0.770; 95% CI, 0.596–0.996; *P* = 0.046; and HR, 0.163; 95% CI, 0.022–0.998; *P* = 0.049, respectively) and lobectomy plus systematic LN dissection or BML with better DFS (HR, 0.737; 95% CI, 0.553–0.983; *P* = 0.038 and HR, 0.468; 95% CI, 0.073–0.978; *P* = 0.047, respectively) compared with that of lobectomy with selected LN biopsy or sampling only. In addition, advanced age, bronchus invasion, and pathological IB stage were identified as being negatively correlated with OS. Good-to-moderate differentiation, adjuvant therapy, and the presence of epithelial growth factor receptor (EGFR) mutations were positively correlated with OS. Patients with squamous cell carcinoma (SCC) and an earlier pathological stage had better DFS than those with other pathological subtypes and a later pathological stage, respectively (Table 3). A similar Cox regression analysis was also performed in the Group Sublobe, and the result is shown in Supplemental Table 2.

5. Comparison of survival among specific groups after PSM

**Figure 5:**
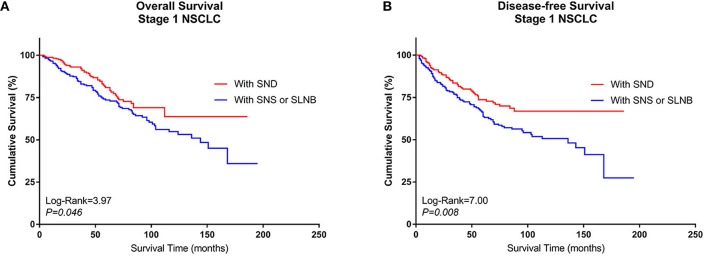
Kaplan–Meier curves of survival estimates for the PSM cohort who underwent lobectomy with SND or with SNS or SLNB. **(A)** Overall survival data for patients who underwent lobectomy with SND, SNS, or SLNB for stage I NSCLC. **(B)** Disease-free survival of patients who underwent lobectomy with SND, SNS, or SLNB for stage I NSCLC. NSCLC, non-small-cell lung cancer; SND, systematic nodal dissection; SNS, systematic nodal sampling; SLNB, selected lymph node biopsy.

After PSM, pairs were formed between Group A and Group B. A total of 468 patients (234 pairs) were included in the PSM analysis, and the baseline characteristics of these patients are summarized in Supplemental Table 3. A Kaplan–Meier survival analysis and log-rank comparison revealed that Group B had better OS (log rank = 3.97, *P* = 0.046) and DFS (log rank = 7.00, *P* = 0.008) than Group A (Figures 5A,B).

**Table 3:** Univariable and multivariable Cox regression analysis for Group Lobe of stage 1 NSCLC patients (*n* = 1,292).

	**Overall survival**				**Disease-free survival**			
	**Univariable analysis**		**Multivariable analysis**		**Univariable analysis**		**Multivariable analysis**	
**Variables**	**HR (95% CI)**	***P*-value**	**HR (95% CI)**	***P-*value**	**HR (95% CI)**	***P*-value**	**HR (95% CI)**	***P*-value**
**Sex**								
Female	Reference		Reference		Reference			
Male	1.559(1.212–2.006)	<0.001	1.261(0.937–1.696)	0.126	1.191(0.966–1.468)	0.102		
**Age(years)**	1.030(1.018–1.042)	<0.001	1.027(1.015–1.039)	<0.001	1.010(1.000–1.020)	0.040	1.009(0.999–1.019)	0.081
**Histology**								
SCC	Reference				Reference		Reference	
Non-SCC	0.867(0.673–1.117)	0.269			1.294(1.008–1.660)	0.043	1.440(1.109–1.871)	0.006
**Differentiation**								
Poor–None	Reference		Reference		Reference			
Well–Moderate	0.687(0.549–0.861)	<0.001	0.748(0.591–0.946)	0.015	0.836(0.683–1.023)	0.082		
**Tumor size(cm)**	1.269(1.128–1.427)	<0.001	1.070(0.928–1.234)	0.350	1.203(1.086–1.332)	<0.001	1.090(0.968–1.227)	0.155
**Smoking history**								
No	Reference		Reference		Reference			
Yes	1.385(1.106–1.733)	0.004	1.070(0.928–1.394)	0.615	1.230(1.009–1.501)	0.041		
**Pathological stage**								
1A1	Reference		Reference		Reference		Reference	
1A2	2.347(0.835–6.595)	0.105	2.19(0.771–6.220)	0.141	2.803(1.005–7.817)	0.049	2.016(0.854–4.757)	0.110
1A3	2.449(0.869–6.902)	0.090	2.040(0.703–5.924)	0.190	4.211(1.524–11.635)	0.006	2.870(1.205–6.835)	0.017
1B	3.544(1.318–9.529)	0.012	2.999(1.051–8.442)	0.038	5.277(1.967–14.158)	0.001	3.418(1.471–7.947)	0.004
**Bronchus invasion**								
No	Reference		Reference		Reference			
Yes	1.286(1.010–1.638)	0.042	1.350(1.047–1.741)	0.021	1.077(0.864–1.344)	0.510		
**Adjuvant therapy**								
No	Reference		Reference		Reference			
Yes	0.701(0.497–0.988)	0.042	0.668(0.469–0.952)	0.025	1.074(0.822–1.404)	0.610		
**Surgical approach**								
Lobectomy	Reference				Reference			
Biolobectomy	0.619(0.256–1.498)	0.287			0.866(0.447–1.677)	0.669		
Pneumonectomy	1.233(0.549–2.769)	0.611			1.005(0.476–2.122)	0.990		
**EGFR mutation**								
Negative	Reference		Reference		Reference			
Positive	0.430(0.241–0.767)	0.004	0.506(0.279–0.920)	0.025	1.188(0.831–1.697)	0.345		
**ALK mutation**								
Negative	Reference				Reference			
Positive	0.738(0.101–5.374)	0.738			1.805(0.661–4.930)	0.250		
**LN dissection group**								
Group A	Reference		Reference		Reference		Reference	
Group B	0.637(0.462–0.879)	0.006	0.668(0.483–0.923)	0.015	0.674(0.506–0.897)	0.007	0.737(0.553–0.983)	0.038
Group C	0.712(0.553–0.916)	0.008	0.770(0.596–0.996)	0.046	0.893(0.712–1.121)	0.330	0.956(0.761–1.201)	0.700
Group D	2.033(0.992–4.164)	0.053	1.649(0.791–3.436)	0.182	2.133(1.084–4.198)	0.028	1.842(0.923–3.673)	0.083
Group E	0.152(0.021–0.967)	0.048	0.163(0.022–0.998)	0.049	0.440(0.154–0.891)	0.037	0.468(0.073–0.978)	0.047

*ALK, anaplastic lymphoma kinase; CI, confidence interval; cm, centimeter; EGFR, epidermal growth factor receptor; HR, hazard ratio; LN, lymph node; NSCLC, non-small-cell lung cancer; SCC, squamous cell carcinoma*.

Multivariable Cox regression analysis also revealed that lobectomy plus selective LN biopsy or sampling was independently associated with worse OS (HR, 1.305; 95% CI, 1.008–1.877; *P* = 0.050) and DFS (HR, 1.417; 95% CI, 1.020–1.968; *P* = 0.038) than other combinations. Additionally, advanced age and poor differentiation without adjuvant therapy were identified as being negatively correlated with OS. Only lobectomy plus systematic LN dissection was positively correlated with DFS (Table 4).

**Table 4:** Univariable and multivariable Cox regression analysis for stage I NSCLC patients in the propensity score matched cohort who underwent lobectomy with SND or with SNS or SLNB.

	**Overall survival**				**Disease-free survival**			
	**Univariable analysis**		**Multivariable analysis**		**Univariable analysis**		**Multivariable analysis**	
**Variables**	**HR (95% CI)**	***P*-value**	**HR (95% CI)**	***P*-value**	**HR (95% CI)**	***P*-value**	**HR (95% CI)**	***P*-value**
**Sex**								
Female	Reference				Reference			
Male	1.317(0.902–1.924)	<0.154			1.071(0.767–1.495)	0.688		
**Age(years)**	1.030(1.013–1.048)	0.001	1.025(1.008–1.042)	0.004	1.011(0.996–1.026)	0.170		
**Histology**								
SCC	Reference				Reference			
Non-SCC	0.954(0.655–1.391)	0.809			1.407(0.959–2.063)	0.081		
**Differentiation**								
Poor–None	Reference		Reference		Reference			
Well–Moderate	0.671(0.476–0.944)	0.022	0.685(0.483–0.970)	0.033	0873(0.633–1.203)	0.406		
**Tumor size(cm)**	1.336(1.110–1.608)	0.002	1.243(0.997–1.550)	0.053	1336(1.130–1.579)	0.001	1.139(0.941–1.379)	0.182
**Smoking history**								
No	Reference				Reference			
Yes	1.201(0.849–1.700)	0.301			1.109(0.802–1.532)	0.532		
**Pathological stage**								
1A1	Reference		Reference		Reference		Reference	
1A2	5.641(0.748–42.538)	0.093	5.061(0.664–38.599)	0.118	2.780(0.639–12.094)	0.173	2.399(0.545–10.557)	0.247
1A3	4.879(0.641–37.123)	0.126	3.886(0.491–30.786)	0.199	3.194(0.734–13.896)	0122	2.335(0.516–10.569)	0.271
1B	7.968(1.111–57.166)	0.039	5.143(0.674–39.262)	0.114	5.552(1.372–22.471)	0.016	3.868(0.898–16.659)	0.069
**Bronchus invasion**								
No	Reference				Reference			
Yes	1.199(0.829–1.735)	0.334			0.896(0.623–1.289)	0.555		
**Adjuvant therapy**								
No	Reference		Reference		Reference			
Yes	0.415(0.203–0.849)	0.016	0.410 (0.198–0.847)	0.016	0.939(0.574–1.536)	0.801		
**EGFR mutation**								
Negative	Reference				Reference			
Positive	0.414(0.152–1.124)	0.084			1.197(0.666–2.153)	0.548		
**ALK mutation**								
Negative	Reference				Reference			
Positive	0.917(0.521–1.617)	0.766			1.500(0.357–6.312)	0.580		
**LN dissection group**								
SND	Reference		Reference		Reference		Reference	
SNS or SLNB	1.435(1.003–2.053)	0.048	1.305(1.008–1.877)	0.050	01.546(1.115–2.144)	0.009	1.417(1.020–1.968)	0.038

*ALK, anaplastic lymphoma kinase; CI, confidence interval; cm, centimeter; EGFR, epidermal growth factor receptor; HR, hazard ratio; LN, lymph node; NSCLC, non-small-cell lung cancer; SCC, squamous cell carcinoma; SLNB, selected lymph node biopsy; SND, systematic nodal dissection; SNS, systematic nodal sampling*.

## Discussion

The extent of tumor resection and LN dissection are important elements in the treatment of resectable early-stage NSCLC (15, 16). Some previous studies demonstrated that sublobectomy results in similar survival outcomes to lobectomy in early-stage NSCLC patients (5, 17). However, other studies have reached different conclusions. In one study, despite adjustment for patient- and tumor-related characteristics, sublobectomy was associated with worse survival, even for stage IA patients (6). Furthermore, in another study, patients undergoing sublobectomy were more likely to have inadequate lymphadenectomies and positive margins than those undergoing lobectomies (18). The results of our study suggest that for surgically resectable stage I NSCLC, most patients undergo lobectomy and that patients who undergo sublobectomy are older and have more comorbidities, smaller tumors, and worse survival.

For LN dissection, the standard model for early-stage NSCLC remains controversial even among different guidelines. The National Comprehensive Cancer Network (NCCN) guidelines recommend that for clinical node-negative stage I NSCLC and patients who underwent sublobectomies, SNS or selected LN biopsy is also acceptable (4). The American College of Chest Physicians (ACCP) guidelines note that for preoperative stage I patients, if they are intraoperatively determined to be node negative, selective LN sampling and dissection are both recommended (19). The European Society of Thoracic Surgeons (ESTS) guidelines recommend SND or sampling for all lung cancer patients. For peripheral T1 SCC, L-SND is also acceptable (3).

Some studies suggested that SND was associated with more accurate staging and better survival, although it results in longer operative times, greater intraoperative blood loss, and recurrent nerve injury; however, the results are inconsistent among studies (20). The results of the American College of Surgery Oncology Group Z0030 trial demonstrated that if systematic sampling of the mediastinal and hilar LNs returns a negative result, mediastinal LN dissection does not improve survival, although it can provide a more accurate staging (9, 10). However, in contrast to the Z0030 trial, which included some pN1 or pN2 and other pIIIA or pIIIB patients, we focused only on pN0 and stage I patients. Moreover, we not only compared SND with SNS by PSM but also more accurately included L-SND and BML in our analysis. All of these may cause a different result between our result and the Z0030 trial result. We found that SND and BML were associated with better OS and DFS than other modalities. Regarding the controversy of LN dissection in stage I patients, the guidelines concentrate mainly on the survival outcome difference between SND and SNS. In our study, a PSM comparison was performed between these two groups, which had not been performed in the Z0030 trial. The results revealed that even after PSM, those who underwent SND had significantly better OS and DFS.

Previous studies also demonstrated that the lymphatic drainage of lung cancer might be lobe specific (21), which provides the basis for L-SND. Some studies suggested that L-SND proved to be as effective as SND (22), but the rate of detection of pN2 was significantly higher in the SND group (12). Our study showed that a significant difference was not found in OS between L-SND and SND. However, compared with SND, L-SND resulted in a worse DFS.

Our study also investigated the relationships between different types of lymphadenectomy in sublobectomy and survival outcomes. However, there were no significant differences between subgroups; this result was attributed to the small sample size. However, other studies found that LN removal appears to decrease locoregional recurrence and may be associated with a survival benefit, while it does not increase morbidity or the length of the hospital stay (23).

Several studies have also demonstrated an association between an increased number of dissected LNs following resection for NSCLC and better long-term survival rates. They indicated that examining a greater number of LNs in patients with stage I NSCLC treated with resection increases the likelihood of proper staging and affects patient outcomes (15). A more recent analysis identified a significant reduction in the mortality rate among patients with at least 16 dissected LNs and clinically certified node-negative NSCLC (24). In the current study, the numbers of resected LNs varied among groups. A greater number of resected LNs was associated with a better survival outcome. Patients who underwent lobectomy with BML may be associated with the greatest number of LNs resected in this subgroup (42.71 ± 21.3).

In our study, we specifically targeted stage I lung cancer and explored the associations between long-term survival and different extents of tumor resection and lymphadenectomy models. Meanwhile, we aimed to clarify which type of lymphadenectomy should be considered a recommended procedure for the surgical treatment of stage I NSCLC. We found that for patients who underwent lobectomy, long-term survival was associated with the type of lymphadenectomy. It is clear that patients who underwent only lobectomy had the worst OS and DFS, and BML resulted in the best OS. For DFS, BML, or SND was associated with better outcomes than that of other subgroups.

These results may be attributed to the following reasons; First, LN involvement can occur in patients with any tumor size (25), and metastasis and micrometastasis can be missed in some cases (26). Therefore, potential metastases may remain if the patient undergoes a sublobectomy or inadequate lymphadenectomy. Second, SND can resect all the possible metastatic tissue, and it can harvest more LNs, which could enhance staging accuracy (24). BML performed better in this respect; thus, it resulted in the best rates of OS and DFS. This may indicate that extended LN dissection may have survival benefits in not only advanced stage NSCLC but also stage I NSCLC. Third, it has been reported that more stringently defined mediastinal LNs are associated with better separation in the prediction of survival. BML can provide the most thorough nodal examination among the different types of lymphadenectomy, which may explain the better outcomes observed among patients who underwent BML and were declared node negative (16).

This study has several limitations of note. Given the retrospective nature of this single-institution study, selection bias is inherent in our study population. In addition, postoperative complications were not considered in our series.

## Conclusion

In conclusion, our study shows that lobectomy with more systematic and complete LN dissection, such as BML or SND, is associated with better survival outcomes in stage I NSCLC patients. However, due to the small sample size, the efficacy of BML still needs to be further verified by a large-scale randomized clinical trial. We recommend lobectomy with SND as the recommended procedure even for stage I NSCLC patients to improve their prognosis. BML is also recommended if it is technically feasible. Despite the use of appropriate statistical methods, our study has some inevitable limitations; more evidence from large-sample-size, multicenter prospective or real-world studies is warranted.

## Data Availability

All datasets generated for this study are included in the manuscript and/or the Supplementary Files.

## Ethics Statement

This retrospective study was approved by the Institute Research Medical Ethics Committee of Sun Yat-sen University Cancer Center. The reference number is B2018-011. All patients included in this research gave written informed consent according to the Research Ethics Committee of Sun Yat-Sen University Cancer Center.

## Author Contributions

LZ was responsible for conception and design of the study. WW, DC, and KX analyzed and interpreted the patient data and were major contributors to the writing of the manuscript. YC, XZ, and YW collected and assembled the data. ZH, XY, GW, and RZ performed the follow-up survey. All authors read and approved the final manuscript.

### Conflict of Interest Statement

The authors declare that the research was conducted in the absence of any commercial or financial relationships that could be construed as a potential conflict of interest.

## Abbreviations

ACCP: American College of Chest Physicians
AJCC: American Joint Committee on Cancer
ALK: anaplastic lymphoma kinase
BML: bilateral mediastinal lymphadenectomy
CIs: confidence intervals
DFS: disease-free survival
EGFR: epidermal growth factor receptor
ELND: extended lymph node dissection
ESTS: The European Society of Thoracic Surgeons
HRs: hazard ratios
KM curves: Kaplan–Meier curves
LN: lymph node
L-SND: lobe-specific systematic node dissection
NCCN: National Comprehensive Cancer Network
NSCLC: non-small-cell lung cancer
OS: overall survival
PSM: propensity score matched
SLNB: selected lymph node biopsy
SND: systematic nodal dissection
SNS: systematic nodal sampling.
