# Supplementary material for: Impact of Different Types of Lymphadenectomy Combined With Different Extents of Tumor Resection on Survival Outcomes of Stage I Non-small-cell Lung Cancer: A Large-Cohort Real-World Study
Source: Front Oncol. 2019 Jul 24;9:642. doi: 10.3389/fonc.2019.00642 (PMC6668052; doi:10.3389/fonc.2019.00642)
Supplement: Supplementary file 1 [file Table_1.DOCX]

| **Supplement table 1.** **Distribution of the clinicopathologic characteristics stratified by gr****oup in Group Sub-lobe (n=44)** | | | | | |
| --- | --- | --- | --- | --- | --- |
| **characteristic** | **Total** | **Group 6 (n=3)** | **Group 7 (n=13)** | **Group 8 (n=28)** | **P** |
| **Gender** |  |  |  |  | 0.345 |
| male | 33(75.0) | 2（66.7） | 8（61.5） | 23（82.1） |  |
| female | 11(25.0) | 1（33.3） | 5（38.5） | 5（17.9） |  |
| **Age（years）** |  |  |  |  | 0.878 |
| Mean±SD | 66.73±11.9 | 63.67±7.5 | 67.62±9.6 | 66.64±13.4 |  |
| Median（min, max) | 68（32,84） | 64（56,71） | 68（50,81） | 69（32,84） |  |
| **Year of procedure** |  |  |  |  | 0.617 |
| 1999-2002 | 0（0.0） | 0 | 0 | 0 |  |
| 2003-2006 | 0（0.0） | 0 | 0 | 0 |  |
| 2007-2010 | 10（22.7） | 0 | 3（23.1） | 7（25.0） |  |
| 2011-2014 | 34（77.3） | 3（100.0） | 10（76.9） | 21（75.0） |  |
| **Histology** |  |  |  |  | 0.811 |
| Non-squamous cell carcinoma | 40 (90.9) | 3（100.0） | 12（92.3） | 25（89.3） |  |
| Squamous cell carcinoma | 4 (9.1) | 0（0） | 1（7.7） | 3（10.7） |  |
| **Cell differentiation** |  |  |  |  | 0.746 |
| Poor-None | 17（38.6） | 1（33.3） | 4（30.8） | 12（42.9） |  |
| Well-Moderate | 27（61.4） | 2（66.7） | 9（69.2） | 16（57.1） |  |
| **Tumor size (cm)** |  |  |  |  | 0.927 |
| Mean±SD | 2.20±0.734 | 2.33±1.5 | 2.15±0.8 | 2.21±0.6 |  |
| Median（min, max) | 2（1,4） | 2（1,4） | 2（1,3） | 2（1,3） |  |
| **Smoking history** |  |  |  |  | 0.865 |
| Yes | 16（36.4） | 1（33.3） | 4（30.8） | 11（39.3） |  |
| No | 28（63.6） | 2（66.7） | 9（69.2） | 17（60.7） |  |
| **Pathological T category** |  |  |  |  | 0.604 |
| T1a | 5（11.4） | 1（33.3） | 1（7.7） | 3（10.7） |  |
| T1b | 13（29.5） | 0 | 5（38.5） | 8（28.6） |  |
| T1c | 7（15.9） | 0 | 3（23.1） | 4（14.3） |  |
| T2a | 19（43.2） | 2（66.7） | 4（30.8） | 13（46.4） |  |
| **Pathological stage** |  |  |  |  | 0.604 |
| I A1 | 5（11.4） | 1（33.3） | 1（7.7） | 3（10.7） |  |
| I A2 | 13（29.5） | 0 | 5（38.5） | 8（28.6） |  |
| I A3 | 7（15.9） | 0 | 3（23.1） | 4（14.3） |  |
| I B | 19（43.2） | 2（66.7） | 4（30.8） | 13（46.4） |  |
| **Adjuvant therapy** |  |  |  |  | 0.617 |
| Yes | 8（18.2） | 1（33.3） | 1（7.7） | 6（21.4） |  |
| No | 36（81.8） | 2（66.7） | 12（92.3） | 22（78.6） |  |
| **Tumor location** |  |  |  |  | 0.296 |
| LUL | 17（38.6） | 1（33.3） | 7（53.8） | 9（32.1） |  |
| LLL | 2（4.5） | 1（33.3） | 0 | 1（3.6） |  |
| RUL | 13（29.5） | 1（33.3） | 2（15.4） | 10（35.7） |  |
| RML | 4（9.1） | 0 | 1（7.7） | 3（10.7） |  |
| RLL | 8（18.2） | 0 | 3（23.1） | 5（17.9） |  |
| **Surgical approach** |  |  |  |  | ＜0.001 |
| Wedge resection | 34（77.3） | 0 | 7（53.8） | 27（96.4） |  |
| Segmentectomy | 10（22.7） | 3（100.0） | 6（46.2） | 1（3.6） |  |
| **Numbers of lymph nodes resected** |  |  |  |  | ＜0.001 |
| Mean±SD | 5.07±10.9 | 33.67±24.7 | 9.38±6.2 | 0 |  |
| Median（min, max) | 0（0,62） | 22（17,62） | 9（1,24） | 0 |  |
| **Treatment after progression of disease** |  |  |  |  | 0.444 |
| Yes | 5（11.4） | 1（33.3） | 1（7.7） | 3（10.7） |  |
| No | 39（88.6） | 2（66.7） | 12（92.3） | 25（89.3） |  |
| **EGFR mutation** |  |  |  |  | 0.332 |
| Negative | 8（18.2） | 0 | 1（7.7） | 7（25.0） |  |
| Positive | 10（22.7） | 1（33.3） | 5（38.5） | 4（14.3） |  |
| Not tested | 26（59.1） | 2（66.7） | 7（53.8） | 17（60.7） |  |
| **ALK mutation** |  |  |  |  | 0.543 |
| Negative | 15（34.1） | 1（33.3） | 6（46.2） | 8（28.6） |  |
| Positive | 0（0.0） | 0 | 0 | 0 |  |
| Not tested | 29（65.9） | 2（66.7） | 7（53.8） | 20（71.4） |  |
|  | *ALK*, anaplastic lymphoma kinase; *cm*, centimeter; *EGFR*, epidermal growth factor receptor; *LUL*, left upper lobe; *LLL*, left lower lobe; *LL*, left lung; *max*, maximum; *min*, minimum; *RUL*, right upper lobe; *RML*, right middle lobe; *RLL*, right lower lobe; *RUML*, right upper-middle lobe; *RMLL*, right middle-lower lobe; *RL*, right lung; *SD*, standard deviation. | | | | |
