# Supplementary material for: Impact of Different Types of Lymphadenectomy Combined With Different Extents of Tumor Resection on Survival Outcomes of Stage I Non-small-cell Lung Cancer: A Large-Cohort Real-World Study
Source: Front Oncol. 2019 Jul 24;9:642. doi: 10.3389/fonc.2019.00642 (PMC6668052; doi:10.3389/fonc.2019.00642)
Supplement: Supplementary file 2 [file Table_2.DOCX]

| **Supplement table 2. Univariable and multivariable Cox regression analysis for the Group Sub-lobe of stage 1 NSCLC patients (n=44)** | | | | | | | | |
| --- | --- | --- | --- | --- | --- | --- | --- | --- |
|  | **Overall survival** | | | | **Disease-free survival** | | | |
|  | **Univariable analysis** | | **Multivariable analysis** | | **Univariable analysis** | | **Multivariable analysis** | |
| **Variables** | **HR （95% CI）** | **P value** | **HR （95% CI）** | **P value** | **HR （95% CI）** | **P value** | **HR （95% CI）** | **P value** |
| **Gender** |  |  |  |  |  |  |  |  |
| Female | Reference |  |  |  | Reference |  |  |  |
| Male | 1.236（0.343-4.445） | 746 |  |  | 0.708（0.241-2.078） | 0.530 |  |  |
| **Age（years）** | 0.999（0.952-1.048） | 0.968 |  |  | 0.977（0.937-1.019） | 0.276 |  |  |
| **Histology** |  |  |  |  |  |  |  |  |
| SCC | Reference |  |  |  | Reference |  |  |  |
| Non-SCC | 0.491（0.107-2.245） | 0.359 |  |  | 0.634（0.143-2.813） | 0.549 |  |  |
| **Differentiation** |  |  |  |  |  |  |  |  |
| Poor-None | Reference |  |  |  | Reference |  | Reference |  |
| Well-Moderate | 0.356（0.114-1.110） | 0.075 |  |  | 0.321（0.112-0.922） | 0.035 | 0.448（0.147-1.361） | 0.156 |
| **Tumor size（cm）** | 1.480（0.641-3.419） | 0.359 |  |  | 1.119（0.538-2.328） | 0.763 |  |  |
| **Smoking history** |  |  |  |  |  |  |  |  |
| No | Reference |  |  |  | Reference |  |  |  |
| Yes | 0.686（0.187-2.516） | 0.570 |  |  | 0.802（0.274-2.352） | 0.688 |  |  |
| **Bronchus invasion** |  |  |  |  |  |  |  |  |
| No | Reference |  |  |  | Reference |  |  |  |
| Yes | 0.042（0.010-121.364） | 0.435 |  |  | 0.733（0.094-5.721） | 0.767 |  |  |
| **Adjuvant therapy** |  |  |  |  |  |  |  |  |
| Yes | Reference |  |  |  | Reference |  | Reference |  |
| No | 1.528（0.411-5.688） | 0.527 |  |  | 4.135（1.451-11.788） | 0.008 | 3.056（0.997-9.363） | 0.051 |
| **Surgical approach** |  |  |  |  |  |  |  |  |
| Wedge resection | Reference |  |  |  | Reference |  |  |  |
| Segmentectomy | 0.035（0.001-45.830） | 0.360 |  |  | 0.249（0.032-1.903） | 0.249 |  |  |
| **EGFR mutation** |  |  |  |  |  |  |  |  |
| Negative | Reference |  |  |  | Reference |  |  |  |
| Positive | 1.343（0.082-21.940） | 0.836 |  |  | 0.307（0.032-2.971） | 0.308 |  |  |
| **ALK mutation** |  |  |  |  |  |  |  |  |
| Negative | Reference |  |  |  | Reference |  |  |  |
| Positive | 0.934（0.242-3.599） | 0.921 |  |  | 1.188（0.372-3.790） | 0.771 |  |  |
| **LN dissection group** |  |  |  |  |  |  |  |  |
| Group F | Reference |  |  |  | Reference |  |  |  |
| Group G | 1.637（0.462-2.879） | 0.953 |  |  | 1.739（1.082-6.692） | 0.788 |  |  |
| Group H | 1.712（0.553-3.916） | 0.955 |  |  | 1.849（1.107-6.746） | 0.877 |  |  |
| *ALK*, anaplastic lymphoma kinase; *CI*, confidence interval; *cm*, centimeter; *EGFR*, epidermal growth factor receptor; *HR*, Hazard ratio; *LN*, lymph node; *NSCLC*, non-small cell lung cancer; *SCC*, squamous cell carcinoma. | | | | | | | | |
