# Supplementary material for: Impact of Different Types of Lymphadenectomy Combined With Different Extents of Tumor Resection on Survival Outcomes of Stage I Non-small-cell Lung Cancer: A Large-Cohort Real-World Study
Source: Front Oncol. 2019 Jul 24;9:642. doi: 10.3389/fonc.2019.00642 (PMC6668052; doi:10.3389/fonc.2019.00642)
Supplement: Supplementary file 3 [file Table_3.DOCX]

| **Supplement table 3.** **The clinicopathologic characteristics of gr****oup 1 and group 2 (n=468)** | | | | |
| --- | --- | --- | --- | --- |
| **Characteristic** | **Total** | **Group 1 (n=234)** | **Group 2 (n=234)** | **P** |
| **Gender** |  |  |  | 0.774 |
| male | 295（63.0） | 149（63.7） | 146（62.4） |  |
| female | 173（37.0） | 85（36.3） | 88（37.6） |  |
| **Age（years）** |  |  |  | 0.479 |
| Mean±SD | 59.72±10.51 | 60.07±11.01 | 59.38±9.99 |  |
| Median（min, max) | 60（23,84） | 61（23,84） | 59（34,80） |  |
| **Year of procedure** |  |  |  | <0.001 |
| 1999-2002 | 33（7.1） | 29（12.4） | 4（1.7） |  |
| 2003-2006 | 69（14.7） | 49（20.9） | 20（8.5） |  |
| 2007-2010 | 134（28.6） | 70（29.9） | 64（27.4） |  |
| 2011-2014 | 232（49.6） | 86（36.8） | 146（62.4） |  |
| **Histology** |  |  |  | 0.199 |
| Non-squamous cell carcinoma | 352（75.2） | 170（72.6） | 182（77.8） |  |
| Squamous cell carcinoma | 116（24.8） | 64（27.4） | 52（22.2） |  |
| **Cell differentiation** |  |  |  | 0.776 |
| Poor-None | 183（39.1） | 90（38.5） | 93（39.7） |  |
| Well-Moderate | 285（60.9） | 144（61.5） | 141（60.3） |  |
| **Tumor size (cm)** |  |  |  | 0.095 |
| Mean±SD | 2.69±0.97 | 2.76±0.96 | 2.61±0.98 |  |
| Median（min, max) | 3（1,4） | 3（1,4） | 2（1,4） |  |
| **Smoking history** |  |  |  | 0.774 |
| Yes | 175（37.4） | 89（38.0） | 86（36.8） |  |
| No | 293（62.6） | 145（62.0） | 148（63.2） |  |
| **Pathological T category** |  |  |  | 0.078 |
| T1a | 25（5.3） | 8（3.4） | 17（7.3） |  |
| T1b | 84（17.9） | 36（15.4） | 48（20.5） |  |
| T1c | 71（15.2） | 35（15.0） | 36（15.4） |  |
| T2a | 288（61.4） | 155（66.2） | 133（56.8） |  |
| **Adjuvant therapy** |  |  |  | 0.385 |
| Yes | 54（11.5） | 24（10.3） | 30（12.8） |  |
| No | 414（88.5） | 210（89.7） | 204（87.2） |  |
| **Tumor location** |  |  |  | 0.977 |
| LUL | 144（30.8） | 73（31.2） | 71（30.3） |  |
| LLL | 80（17.1） | 39（16.7） | 41（17.5） |  |
| LL | 0 | 0 | 0 |  |
| RUL | 108（23.1） | 55（23.5） | 53（22.6） |  |
| RML | 25（5.3） | 13（5.6） | 12（5.2） |  |
| RLL | 106（22.6） | 52（22.2） | 54（23.2） |  |
| RUML | 1（0.2） | 0 | 1（0.4） |  |
| RMLL | 4（0.9） | 2（0.8） | 2（0.8） |  |
| RL | 0 | 0 | 0 |  |
| **Numbers of lymph nodes resected** |  |  |  | <0.001 |
| Mean±SD | 18.16±10.47 | 12.18±7.19 | 24.15±9.79 |  |
| Median（min, max) | 17（1，79） | 11（1,43） | 22（7,79） |  |
| **Treatment after progression of disease** |  |  |  | 0.802 |
| Yes | 76（16.2） | 39（16.7） | 37（15.8） |  |
| No | 392（83.8） | 195（83.3） | 197（84.2） |  |
| **EGFR mutation** |  |  |  | 0.907 |
| Negative | 70（15.0） | 36（15.4） | 34（14.5） |  |
| Positive | 67（14.3） | 32（13.7） | 35（15.0） |  |
| Not tested | 331（70.7） | 166（70.9） | 165（70.5） |  |
| **ALK mutation** |  |  |  | 1.000 |
| Negative | 82（17.5） | 41（17.5） | 41（17.5） |  |
| Positive | 4（0.9） | 2（0.9） | 2（0.9） |  |
| Not tested | 382（81.6） | 191（81.6） | 191（81.6） |  |
|  | *ALK*, anaplastic lymphoma kinase; *cm*, centimeter; *EGFR*, epidermal growth factor receptor; *LUL*, left upper lobe; *LLL*, left lower lobe; *LL*, left lung; *max*, maximum; *min*, minimum; *RUL*, right upper lobe; *RML*, right middle lobe; *RLL*, right lower lobe; *RUML*, right upper-middle lobe; *RMLL*, right middle-lower lobe; *RL*, right lung; *SD*, standard deviation. | | | |
